# Cytology of a seminoma in a koi (*Cyprinus carpio*): a rapid diagnostic tool

**DOI:** 10.1007/s11259-024-10391-3

**Published:** 2024-05-21

**Authors:** Claudio Pigoli, Gabriele Ghisleni, Federico Armando, Valeria Grieco, Andrea Ghidelli, Eleonora Brambilla

**Affiliations:** 1 Laboratorio Di Istologia, Sede Territoriale Di Milano, Istituto Zooprofilattico Sperimentale Della Lombardia E Dell’Emilia-Romagna (IZSLER), Brescia, Italy; 2 BiEsseA Laboratorio Analisi Veterinarie, an Antech Company, Milan, Italy; 3 Department of Pathology, University of Veterinary Medicine, Foundation, Hannover, Germany; 4 Department of Veterinary Medicine and Animal Sciences, University of Milan, Via Dell’Università 6, 26900 Lodi, Italy; 5 Clinica Veterinaria Sant’Emiliano, Brescia, Italy

**Keywords:** Cytology, Koi carp, Seminoma

## Abstract

Koi(*Cyprinus carpio*) is an ornamental variety of common carp frequently kept as pets. Given their long lifespan, neoplasia, albeit uncommon, may occur in these animals, and only a few studies have faced their cytological diagnosis. In the present case, a koi carp was referred to the clinicians due to coelomic swelling. The carp underwent surgery, which revealed an enlargement of both testes. Testicular samples were cytologically and histologically examined. The lesion was diagnosed as a seminoma since it was composed of round, large, atypical, and often multinucleated cells with round central nuclei and moderate cytoplasm. These tumors had the same appearance as seminomas in mammals and should be considered among differential diagnoses when coelomic swelling occurs in koi carp. Seminomas in koi carp are diagnosed histologically, but cytology, a rapid and cheap exam executable in all veterinary clinical facilities, could be a relevant preliminary diagnostic tool that may influence the entire diagnostic process.

## Introduction

Koi (*Cyprinus carpio*) is an ornamental variety of common carp frequently kept as pets in domestic ponds worldwide. Due to their tame behavior and long lifespan, they have high economic and emotional value as pets (Sirri et al. 2021).

To date, only a few studies have reported the prevalence of tumors in koi (Sirri et al. 2021). Some tumors in fish have been attributed to genetic factors (Meierjohann and Schartl 2006; Nairn et al. 1996); others were associated with viral infection (Hanson et al. 2011; Coffee et al. 2013) or with environmental contamination (Fabacher and Baumann 1985; Baumann et al. 1990; Harshbarger and Clark 1990). However, since the presence of tumors in koi populations includes just sporadic case reports of tumors worldwide (Knüsel et al. 2007; Sirri et al. 2010, 2012; Stegeman et al. 2010), data about the prevalence or significance of neoplastic lesions in koi are still missing (Ferraro et al. 2024). Among the tumors, neoplastic lesions of internal organs are particularly represented, with case numbers increasing over the last years (Ott Knüsel et al. 2015).

In cyprinids, a high prevalence of spontaneous gonadal neoplasms has been reported in hybrids of goldfish Carassius auratus L. × common carp Cyprinus carpio L. (Sonstegard 1977; Leatherland and Sonstegard 1978; Dickman and Steele 1986; Granado-Lorencio et al. 1987; Down and Leatherland 1989; Sirri et al. 2010).

According to data collected by breeders and examination of various previous documents, ovarian neoplasms in ornamental koi Cyprinus carpio L. are similar to those described in wild goldfish × carp hybrids: they are common in sexually mature females and originate from the ovary, although the cellular origin is often difficult to determine (Groff 2004).

Of the gonadal tumors described in the literature in fish, ovarian tumors are the most reported, while testicular tumors are more rarely described (H. Schmidt-Posthaus & R. Knüsel unpubl. Data; Sirri et al. 2010). In particular, in 2010 a case of spontaneous testicular tumor was described by Sirri et al. and was classified by histological and immunohistochemical investigation according to the WHO International Histological Classification of the Tumors of the Genital System in use for mammals as diffused and classical seminoma (Kennedy et al. 1998; Sirri et al. 2010). Despite what is already reported in the literature, there are no cases in which the application of cytology has been used as a diagnostic tool to obtain an initial diagnosis at the surgical site to be confirmed later by diagnostic methods such as histology or immunohistochemistry. Therefore, the present study is the first case in which cytology is used for this purpose.

## Case report

In the present case, a koi was presented to the referring veterinarian due to coelomic swelling. The carp underwent surgery, which revealed an enlargement of removed testes. Testes measured 19 x 10.5 x 9 cm and 20.5 x 6 x 3.5 cm were cocoonlike and yellow whitish. Some cytological samples were performed. Cytological samples consisted of imprints obtained by placing the mass on the slide and stained with Diff Quick stain. Then, testicular samples were collected, fixed in 10% neutral buffered formalin, and serial sections were obtained and stained with Hematoxylin-Eosin (H&E) for histological examination as previously described (Armando et al. 2021).

**Fig. 1:**
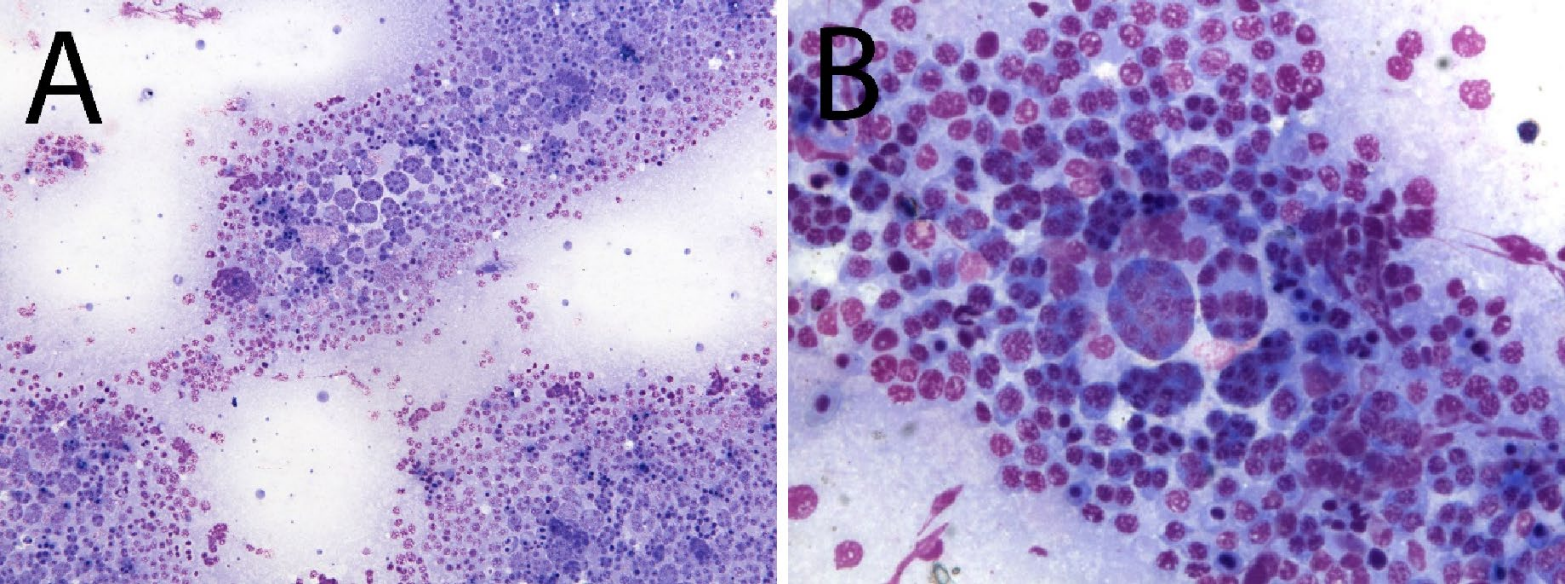
Figure demonstrates atypical or neoplastic cells sampled from the testis. The cytological appearance of the lesion (Diff Quick, **A**: 20X, **B**: 60X): atypical cells admixed with occasional small mature lymphocytes embedded in a moderate amount of bluish fluid. Most cells were round to oval with distinct margins, intermediate to high nucleus-cytoplasmic ratio, and moderate to scant homogeneous bluish cytoplasm. Bi- and multinucleated atypical cells were also present

The cytological samples were highly cellular, poorly hemodiluted, and composed of a mixed cellular population mainly consisting of atypical cells admixed with occasional lymphocytes and embedded in a moderate amount of bluish fluid (Fig. 1A).

Most cells consisted of round to oval cells with distinct margins, intermediate to high nucleus-cytoplasmic ratio, and moderate to scant homogeneous bluish cytoplasm. Nuclei were round and eccentric, with coarsely dispersed chromatin and occasionally a single prominent nucleolus. Anisocytosis and anisokariosis were moderate, and mitoses were rare. Numerous bi- and multinucleated atypical cells were also observed (Fig. 1B).

**Fig. 2:**
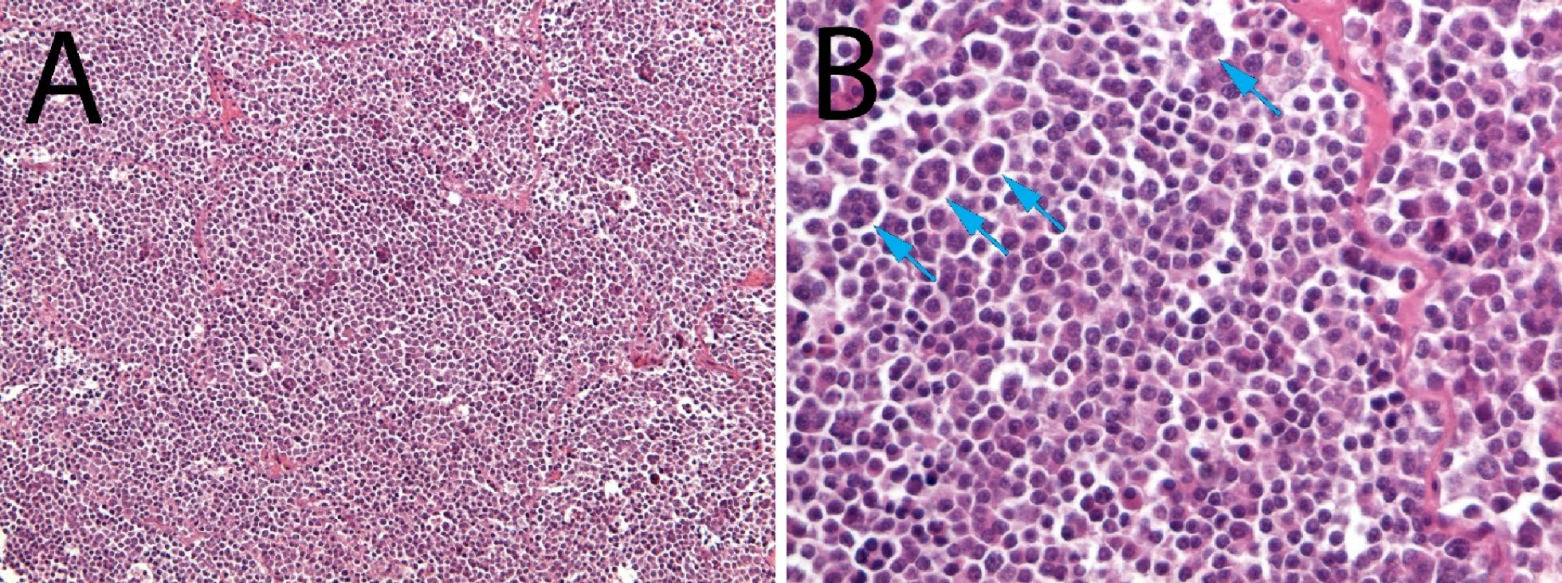
Figure demonstrates atypical or neoplastic cells sampled from the testis, histological appearance of the lesion (Hematoxylin–Eosin, **A**: 20X, **B**: 40X). Neoplasia was composed of round cells arranged in sheets supported by a thin fibrovascular stroma. Cells were round with abundant eosinophilic to amphophilic homogeneous cytoplasm and round to oval central large nuclei. Moreover, numerous multinucleated neoplastic cells were also visible (arrows). Unfortunately, the carp died during the surgical procedure

Histologically, the parenchyma of both testicles was diffusely effaced and replaced by a densely cellular, multilobular, poorly demarcated, unencapsulated, infiltrative neoplasm. The neoplasm was composed of round cells arranged in sheets and small clusters, which were variably supported by a thin fibrovascular stroma. Atypical cells were round from 25 to 30 µm in diameter with abundant eosinophilic to amphophilic homogeneous cytoplasm and moderate to high nuclear-cytoplasmic ratio. Nuclei were round to oval, ranged from 15 to 25 µm in diameter, central to paracentral with finely stippled and often marginated chromatin and one occasionally visible eosinophilic nucleolus. Anisocytosis and anisokariosis were moderate to high, and there were 9 mitoses in 2.37 mm^2^; numerous multinucleated neoplastic cells were also present. Intratumoral necrotic areas were multifocally observed (Fig. 2).

## Discussion

Considering the spread of koi, and their value as pet animals, an improvement in the veterinary diagnostic algorithmis needed. Neoplastic diseases are described in these animals, and gonadal tumors should be considered in cases of coelomic swelling in koi. In the literature, gonadal tumors in koi are described but as exposed in a recent study by Ott Knüsel et al. conducted in 2016, these tumors are mostly represented by ovarian predominantly sex cord stroma tumors, whereas tumors originating from germ cells account for only 2.5 % of coelomic neoplasms being relatively rare although reported in the literature (Ott Knüsel et al. 2016). The causes of the onset of these tumors are not yet known. However, the request for particular color varieties has increased the selection and inbreeding of the species; thus, a genetic predisposition has been suggested (Ott Knüsel et al. 2015). Moreover, since few studies exist on these tumors, environmental factors such as toxic compounds, or viral causes cannot be excluded (Sirri et al. 2021). In fish, organic pollutants are often absorbed through the gills and skin, and accumulate in lipid-rich tissues, such as liver, brain, gonads, and hypodermal lipid storages. (Baines et al. 2021) In particular, exposure to substances such as: PAH (7,12-Diniethylbenz[a]anthracene), Ethlynitrosourea, N-methyl-N’-nitro-N-nitrosoguanidine (MNNG), PCB’s, pesticides (ß-endosulfan and α-endosulfan), hydrocarbons (oil), heavy metals, are known to be related to the occurrence of gonadal tumors in fish, particularly seminomas and dysgerminomas (Baines et al. 2021; Bunton and Wolfe 1996; Spitsbergen et al. 2000a, 2000b).

In the present case, the neoplasia described was composed of cells that resemble normal germinal epithelium and have oval nuclei, straight cell borders and distinct Golgi complex,. These aspects, together with the presence of intercellular bridges, as seen in normal germinal cells, are present in seminomas (Maxie 2015). These histological and cytological features allow the clear distinction of these tumors from the differential diagnoses of other testicular tumors such as interstitial or Sertoli cell tumors. Given the histological and cytological findings observed in this case, the present neoplasia was diagnosed as a spontaneous seminoma.

Seminomas in fish are reported in literature and are described as tumors composed of typical germ cells similar to those from humans and equivalent mammalian tumors. This enables the comparative oncologist to classify fish tumors on the same bases as mammal tumors (Masahito et al. 1988).

According to the WHO International Histological Classification of the Tumors of the Genital System of Domestic Animals (Kennedy et al. 1998), the present seminoma could be classified as diffuse, given the lobular arrangement of neoplastic cells divided by a stromal component infiltrated by lymphocytes suggested a similarity with the diffuse form. However, the high malignancy of our seminoma and the probable origin of the neoplastic cells from undifferentiated seminal cells suggest that the present seminoma is ascribable to the classical type, according to the WHO classification of testicular tumors in humans (Mostofi and Sesterhenn 1998).

As occurs for mammals, gonadal tumors are diagnosed histologically supported by cytological examination. However, in order to confirm the cytological and histological diagnosis and, above all, to classify seminomas according to classifications in human and veterinary medicine, an immunohistochemical panel, tested in a previous case of seminoma in a koi described by Sirri et al. in 2010 together with the PAS staining, is available (Sirri et al. 2010). This panel included several markers including in particular cytokeratin, vimentin, c-KIT, placental alkaline phosphatase (PLAP), and neuronspecific enolase (NSE), revealing an immunoreactivity of seminomatous germ cells to vimentin, PLAP, and c-KIT, but not to NSE and cytokeratin (Feitz et al. 1987; Foster and Ladds 2007; Grieco et al. 2007; Sirri et al. 2010). PLAP, which is produced ectopically by a variety of malignant tumors including human seminoma, was found to be a specific antibody for neoplastic cells of a classical histotype (Lange et al. 1982; Grieco et al. 2007). c-KIT, which is normally expressed by germ cells, has been validated as a marker to distinguish seminoma from Sertoli cell tumors, as it is also expressed by undifferentiated neoplastic seminal cells (Grieco et al. 2010; Yu et al. 2009; Sirri et al. 2010). However, the use of mammalian antibodies in fish tissues has certain limitations related to their specificity. In addition the immunohistochemical panel is useful for classifying the neoplasm whereas, the cytologic and histopathologic diagnosis is itself quite accurate given the particularity of the neoplasm and its very different appearance from the main differential diagnoses. Therefore, the cytological examination, which is quick, inexpensive and can be performed at the surgical site, is an excellent first-stage diagnostic tool. Little is known about the prognosis of these neoplasms as there is only one case report in the literature of a black sea bass in which surgery was performed to remove a seminoma diagnosed by histological examination. In that case, surgery was successful, as an improvement in the patient’s vital parameters and the absence of a recurrence of the neoplasm during follow-up diagnostic investigations eight weeks after surgery have been described (Weisse et al. 2002). The cases described in the literature concerning surgical procedures for the removal of seminomas in koi and their post-operative prognosis are rare. The present report does not provide any further information in this respect as the koi died during the surgical procedure. There are currently studies in the literature in which new anaesthetic protocols are being tested with the aim of reducing the already high anaesthesiological risk in fish. This risk depends on several factors such as the sensitivity of these species to anaesthetics, drug dosage, anaesthesia monitoring and post-operative hospitalisation (Gladden et al. 2010).

## Conclusion

Seminomas in koi carp are diagnosed histologically and classified immunohistochemically, but cytology, a rapid and cheap exam executable in all veterinary clinical facilities, could be a relevant preliminary diagnostic tool that may influence the entire diagnostic process.

## Data Availability

No datasets were generated or analysed during the current study.
